# First Universal Newborn Screening Program for Severe Combined Immunodeficiency in Europe. Two-Years' Experience in Catalonia (Spain)

**DOI:** 10.3389/fimmu.2019.02406

**Published:** 2019-10-22

**Authors:** Ana Argudo-Ramírez, Andrea Martín-Nalda, Jose L. Marín-Soria, Rosa M. López-Galera, Sonia Pajares-García, Jose M. González de Aledo-Castillo, Mónica Martínez-Gallo, Marina García-Prat, Roger Colobran, Jacques G. Riviere, Yania Quintero, Tatiana Collado, Judit García-Villoria, Antonia Ribes, Pere Soler-Palacín

**Affiliations:** ^1^Newborn Screening Laboratory, Inborn Errors of Metabolism Division, Biochemistry and Molecular Genetics Department, Hospital Clínic, Barcelona, Spain; ^2^Pediatric Infectious Diseases and Immunodeficiencies Unit, Jeffrey Modell Diagnostic and Research Center for Primary Immunodeficiencies, Hospital Universitari Vall d'Hebron, Universitat Autònoma de Barcelona, Barcelona, Spain; ^3^Immunology Division, Jeffrey Modell Diagnostic and Research Center for Primary Immunodeficiencies, Hospital Universitari Vall d'Hebron, Universitat Autònoma de Barcelona, Barcelona, Spain; ^4^Department of Clinical and Molecular Genetics, Jeffrey Modell Diagnostic and Research Center for Primary Immunodeficiencies, Hospital Universitari Vall d'Hebron, Universitat Autònoma de Barcelona, Barcelona, Spain

**Keywords:** newborn screening, severe combined immunodeficiency, T-cell receptor excision circles, T-cell receptor, T-lymphocytes, stem cell transplantation

## Abstract

Severe combined immunodeficiency (SCID), the most severe form of T-cell immunodeficiency, can be screened at birth by quantifying T-cell receptor excision circles (TRECs) in dried blood spot (DBS) samples. Early detection of this condition speeds up the establishment of appropriate treatment and increases the patient's life expectancy. Newborn screening for SCID started in January 2017 in Catalonia, the first Spanish and European region to universally include this testing. The results obtained in the first 2 years of experience are evaluated here. All babies born between January 2017 and December 2018 were screened. TREC quantification in DBS (1.5 mm diameter) was performed with the Enlite Neonatal TREC kit from PerkinElmer (Turku, Finland). In 2018, the retest cutoff in the detection algorithm was updated based on the experience gained in the first year, and changed from 34 to 24 copies/μL. This decreased the retest rate from 3.34 to 1.4% (global retest rate, 2.4%), with a requested second sample rate of 0.23% and a positive detection rate of 0.02%. Lymphocyte phenotype (T, B, NK populations), expression of CD45RA/RO isoforms, percentage and intensity of TCR αβ and TCR γδ, presence of HLA-DR+ T lymphocytes, and *in vitro* lymphocyte proliferation were studied in all patients by flow cytometry. Of 130,903 newborns screened, 30 tested positive, 15 of which were male. During the study period, one patient was diagnosed with SCID: incidence, 1 in 130,903 births in Catalonia. Thirteen patients had clinically significant T-cell lymphopenia (non-SCID) with an incidence of 1 in 10,069 newborns (43% of positive detections). Nine patients were considered false-positive cases because of an initially normal lymphocyte count with normalization of TRECs between 3 and 6 months of life, four infants had transient lymphopenia due to an initially low lymphocyte count with recovery in the following months, and three patients are still under study. The results obtained provide further evidence of the benefits of including this disease in newborn screening programs. Longer follow-up is needed to define the exact incidence of SCID in Catalonia.

## Introduction

Newborn screening (NBS), initially implemented in the United States in 1961 (1), has been available in most developed countries for decades, originally for the detection and early treatment of phenylketonuria and now for other endocrine and metabolic diseases, mainly using tandem mass spectrometry. DNA-based testing for other diseases has been only recently included in the NBS programs of some countries. Since its initial implementation in Wisconsin in 2008, NBS for severe combined immunodeficiency (SCID) using a T-cell receptor excision circle (TREC) assay has been established worldwide, including in most of the United States (2), Taiwan (3), Israel (4), New Zealand, some Canadian regions (2), Norway (5), and Catalonia (Spain). Previously published results showed an incidence of SCID of around 1 in 56,000 newborns (5, 6) and a high survival rate (92%) after appropriate treatment (2).

SCID, the most severe form of T-cell primary immunodeficiency (PID), includes a group of inherited defects characterized by severe T-cell lymphopenia (TCL). Patients with SCID require prompt clinical intervention to prevent severe life-threatening infections, and several studies have reported significantly improved survival in babies diagnosed at birth (7, 8). Curative treatment is based on hematopoietic stem cell transplantation (HSCT) or gene therapy when available (8). SCID can be screened at birth in a cost-effective way on a large scale through quantification of T-cell receptor excision circles (TRECs) in Guthrie card dried blood spot (DBS) samples (9, 10).

SCID newborn screening can also identify other clinically relevant forms of TCL, which have an overall incidence of 1 in 7,300 newborns (2). These include specific syndromes such as 22q11 deletion (DiGeorge) syndrome, Down syndrome, and CHARGE syndrome among others (11, 12).

TRECs are circular DNA molecules formed by T-cell receptor gene rearrangement during the normal process of T-cell differentiation in the thymus. TRECs do not undergo further replication in dividing cells. Hence, they are stable circular DNA fragments, which are a useful marker of recently formed T-cells, a cell population that is extremely low in newborns with SCID. TREC copy number can be determined using a quantitative PCR-based method with time-resolved fluorescence resonance energy transfer (TR-FRET)-based detection in eluted DNA from routinely collected DBS (9). Kappa-deleting recombination excision circles (KRECs), the circular by-product of B cell immunoglobulin kappa gene rearrangement, have been proposed for a combined TREC-KREC screening approach (13). This could also enable the early detection of patients with severe forms of B cell deficiency, such as X-linked agammaglobulinemia (XLA) (14).

The implementation of screening through TREC assays provides the earliest possible identification and allows for prompt, successful transplantation before infants experience severe infection, organ damage, and, ultimately, death (15). The cost-effectiveness of including SCID in NBS programs has been demonstrated in the United States (16, 17) and Europe (6, 10, 18, 19).

In Catalonia, the NBS program started with phenylketonuria detection in 1969 and congenital hypothyroidism in 1982. Today, around 67,000 newborns per year are screened for 24 diseases (phenylketonuria, congenital hypothyroidism, cystic fibrosis, sickle cell disease, aminoacidopathies, organic acidurias, mitochondrial beta-oxidation disorders, and, lately, SCID). SCID was included in the Catalonian NBS program in January 2017, and data from the first 24 months following its implementation are presented here.

## Materials and Methods

### Population and Data Collection

All consecutive DBS samples received as part of the universal NBS program in Catalonia between January 2017 and December 2018 were analyzed (*n* = 130,903). Samples with the following characteristics were excluded: collection time before 44 h or after 7 days of life, transfusions, poor DNA amplification, and poor quality or blood amount. Ultimately, this study was performed in 129,614 newborns.

Demographics (birth date, date of sample collection, parents' origin, newborn sex, gestational age, and birth weight) were electronically collected. Extremely preterm newborns were defined as those with a gestational age <32 weeks, preterm newborns ≥32 and <37 weeks, and term newborns ≥37 weeks. Low birth weight in term babies was defined as <2,500 g and normal birth weight as ≥2,500 g.

**Figure 1:**
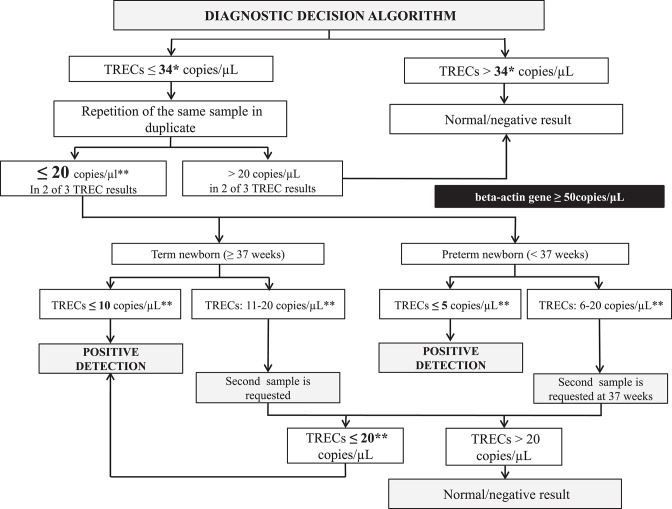
SCID NBS detection decision algorit hm. ^*^The retest cutoff was changed from 34 to 24 copies/μL in 2018. ^**^If beta-actin gene <50 copies/μL a second sample was requested because the sample was considered of unsatisfactory quality. TRECs, T-cell receptor excision circles.

From 1 January to 30 June 2017, newborns (*n* = 33,040) underwent SCID screening as part of a 6-month prospective implementation pilot study that validated our approach. However, in January 2018, we decided to update the decision algorithm (Figure 1), lowering the retest cutoff from 34 to 24 copies/μL. The results from newborns screened in 2018 were then evaluated (*n* = 64,092; 63,393 after applying exclusion criteria).

The study was approved by the Government of Catalonia (*Departament de Salut, Generalitat de Catalunya*) for its NBS program. Specific informed consent was obtained from all individual participants included in the study whose genetic evaluation was needed.

### Sample Testing

Quantification of TRECs in DBS (1.5-mm diameter spot) was performed according to the commercial Enlite Neonatal TREC kit instructions (PerkinElmer, Turku, Finland). The EnLite TREC kit is a combination of PCR-based nucleic acid amplification and time-resolved fluorescence resonance energy transfer (TR-FRET)-based detection. The assay detects two targets simultaneously: TRECs and beta-actin, which is used as the internal control to monitor specimen amplification in each test.

DBS punches were directly inserted in 96-well PCR plates using a Wallac DBS puncher (PerkinElmer). DNA was eluted in the first step, and the second step involved PCR amplification of TRECs and beta-actin as well as hybridization with target sequence-specific probes. The PCR plate was then read in a Victor EnLite fluorometer (PerkinElmer). A full calibration curve with blanks and three DBS calibrators was run in triplicate on each plate. A low-TREC control, a no-TREC control, a high-TREC control, and a blank paper disk (with no blood content) were used as quality controls in each plate. Interpretation of the assay results uses fluorescence counts measured at 615, 665, and 780 nm. Corrected fluorescence counts, TREC responses, and beta-actin responses for all reactions were calculated from the raw fluorescence counts. Calibrator responses were fitted against the ArcSinh transformed concentrations (copies/μL) using unweighted linear regression. The Enlite software generated a calibration curve and the sample and DBS control concentrations (copies/μL) in each run.

### Definition and Interpretation of Results

Before implementing the protocol, the method was verified (repeatability, reproducibility, limit of detection, limit of quantification, sensitivity, and specificity) and these parameters were successfully compared with those stated in the kit insert (Supplementary Material S1). In addition, available NBS samples from children with a known SCID diagnosis in Catalonia in the last 5 years were analyzed as positive controls (*n* = 6; median, range TREC copies/μL: 2, 2–4), as well as five other positive samples from the SCID Newborn Screening Quality Assurance Program-Proficiency Testing Program provided by the CDC (Centers for Disease Control and Prevention, Atlanta, USA).

After reviewing the decision algorithms from other NBS programs with previous experience of this disease, we decided to start the pilot study with the algorithm used by Audrain et al. (6), which had a threshold of >34 copies/μL (Figure 1).

Samples whose TREC value was ≤ 34 copies/μL were retested in duplicate. If two of the three values were ≤ 20 copies/μL, a second sample was requested. Samples with TRECs ≤ 5 copies/μL (preterm infants) or ≤ 10 copies/μL (term newborns) in the first sample (both with beta-actin gene ≥50 copies/μL), as well as analyses with TRECs ≤ 20 copies/μL in the second sample, were considered as positive detection (retest cutoff changed from 34 to 24 copies/μL in 2018). These positive detections were notified to the SCID Clinical Reference Unit (SCID-CRU) to initiate clinical and immunological evaluation.

The retest after the first sample rate (retest rate), requested second sample rate, and SCID-positive detection rate (positive detections) were calculated. Based on these results, the algorithm was reevaluated. Validation of the results was carried out using Specimen Gate (Perkin Elmer) and Nadons (Limit4, Barcelona, Spain) software.

### Initial Clinical and Immunological Assessment at SCID-CRU

**Figure 2:**
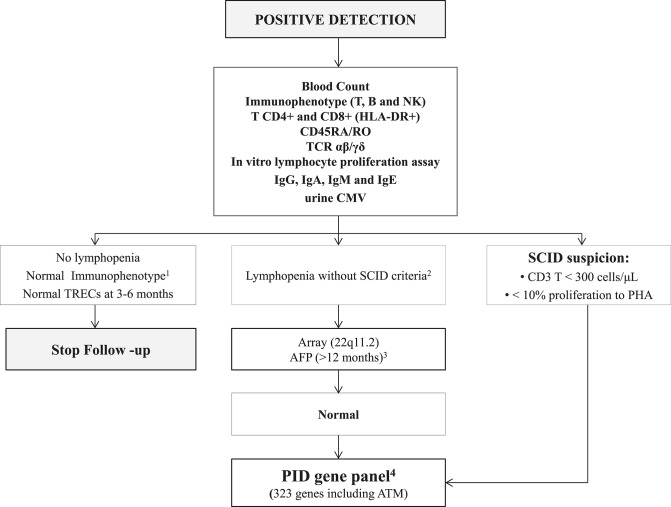
Immunological and genetic protocol in positive cases. ^1^Flow cytometry protocols are shown in Supplementary Material S5; ^2^Lymphopenia and SCID criteria are shown in Supplementary Material S2; ^3^AFP values are not reliable before 12 months of age; therefore, AFP was only studied in patients older than one year lacking an alternative diagnosis; ^4^The list of 323 PID genes is shown in Supplementary Material S3. AFP, alpha-fetoprotein; ATM, ataxia telangiectasia; B, B-cells; CMV, cytomegalovirus; Ig, immunoglobulin; NK, natural killer cells; PID, Primary immunodeficiency; SCID, severe combined immunodeficiency; T, T-cells; TCR, T-cell receptor; TRECs, T-cell receptor excision circles.

Within the first 7 days after detection, all positive cases were referred to SCID-CRU, where clinical and immunological assessment was performed per protocol (Figure 2). Complete family and medical histories were recorded, and a meticulous physical examination was carried out. In addition, psychological support was offered to parents, starting with this first visit.

Several tests were performed: T, B, and NK cell immunophenotyping, expression of CD45RA/RO, TCR αβ, and γδ, HLA-DR+ expression on T lymphocytes, *in vitro* lymphocyte proliferation, and immunoglobulins including IgE. SCID criteria (Supplementary Material S2) were defined as reported by Kwan et al. (2). In cases of non-SCID lymphopenia, a consultation was scheduled with the geneticist for clinical evaluation and array-based comparative genomic hybridization (CGH-array) studies. If these results were normal, a genetic panel covering most PID was performed. We used a custom-designed next-generation sequencing (NGS)-based panel that targets 323 genes (Supplementary Material S3), including most of the known PID-causing genes according to the 2017 IUIS (International Union of Immunological Societies) classification, and other genes recently described as causing PID (20). This panel has been successfully used in our laboratory over the last few years for genetic diagnosis of PID (21).

### Statistical Analysis

Statistical analyses were performed using SPSS software version 23.0 (SPSS Inc., Chicago, Illinois). Given the non-parametric distribution of the data, the Mann–Whitney *U* or Kruskal–Wallis test were used for group comparison analyses (25 and 75th percentiles in brackets). Two-tailed statistical analysis was performed, and differences were considered statistically significant at *p* < 0.05.

## Results

### Demographics

Of the 129,614 newborns screened after applying the exclusion criteria (initial *n* = 130,903), 51.5% were male and 48.5% were female. The median age at sample collection was 50 h of life (interquartile range [IQR], 49–60). Median gestational age was 39 weeks (38–40): 0.6% were extremely preterm newborns (*n* = 830), 5.9% preterm (*n* = 7,561), and 93.5% term newborns (*n* = 121,223). Median birth weight in term newborns was 3,300 g (3,010–3,590) (Table 1).

**Table 1:** Demographic data and TREC values in the study population.

**Sample size**	***n* = 129,614^a^**	**TRECs, copies/μL, median (IQR)**	***p*-value^c^**
All newborns		104 (68-162)	–
Sex			
Male	51.5% (66, 751)	98 (64–152)	<0.05
Female	48.5% (62, 863)	111 (73–173)	
Sample collection, hours, median (IQR)	50 (49–60)	–	–
Gestational age, weeks, median (IQR)	39 (38–40)		
Extremely preterm, <32 weeks, % (*n*)	0.6% (830)	70 (43–127)	<0.05
Preterm, 32-36 weeks, % (*n*)	5.9% (7,561)	96 (62–155)	
Term, ≥37 weeks, % (n)	93.5% (121, 223)	105 (68–162)	
Birth weight in term newborns^b^, grams, median (IQR):	3300 (3,010–3,590)		
Low birth weight, % (*n*)	3.4% (4,385)	94 (61–145)	<0.05
Normal weight,% (*n*)	96.6% (114, 777)	105 (69–163)	

a Sample size after applying exclusion criteria;

b *Term newborns (n = 121, 223)*.

c *Mann-Whitney U or Kruskal-Wallis test were used for group comparisons (p-value calculated at a 5% significance level). IQR, interquartile range*.

Median TREC value in the study population was 104 copies/μL (IQR: 68–162), with 20 copies/μL being the 0.7th percentile. Median TREC values showed statistically significant differences in relation to the patients' sex, gestational age, and birth weight (only assessed in term babies). Median TREC values were 98 (64–152) and 111 (73–173) in males and females, respectively (*p* < 0.05). In relation to gestational age, the median TREC value demonstrated a progressive increase: 70 (43–127), 96 (62–155), and 105 (68–162) copies/μL in extremely preterm, preterm, and term newborns, respectively (*p* < 0.05) (Supplementary Material S4). Regarding birth weight in term newborns, median TREC was 94 (61–145) and 105 (69–163) copies/μL in low and normal birth weight babies, respectively (*p* < .05) (Table 1).

### Cutoff Values and Rates

**Figure 3:**
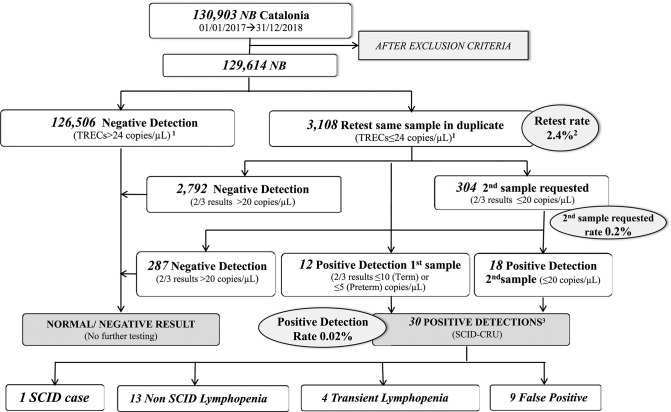
Results of SCID NBS in Catalonia over the study period (2017–2018). ^1^2018 cutoff (2017 cutoff = 34 copies/μL); ^2^2017+2018-Retest rate 2017-Retest rate = 3.34% (cutoff = 34 copies/μL); 2018-Retest rate = 1.4% (cutoff = 24 copies/μL); ^3^Three patients are currently under study. NB, newborns; SCID, severe combined immunodeficiency; SCID-CRU, SCID Clinical Reference Unit.

The overall results from 2017 to 2018 were retest rate 2.4%, requested second sample rate 0.23%, and positive detection rate 0.02% (Figure 3).

With the application of the initial TREC retest cutoff of 34 copies/μL (3rd percentile in the 2017 population), results from the 66,214 samples analyzed during 2017 were as follows: retest rate 3.34% (*n* = 2212), requested second sample rate 0.21% (*n* = 138), and positive detection rate 0.02% (*n* = 16).

After reducing the retest cutoff to 24 copies/μL (1st percentile), the retest rate was 1.4% (*n* = 898). The detection cutoff of 20 copies/μL remained the same (0.7th percentile), and the requested second sample and positive detection rates were similar to those of 2017.

### Positive Detections

There were 30 positive detections in the 129,614 screened newborns: 40% (*n* = 12) were detected in the first sample and 60% (*n* = 18) in the second, with 50% (*n* = 15) of detections occurring in males. There was no history of maternal immunosuppression in any of the positive cases.

**Figure 4:**
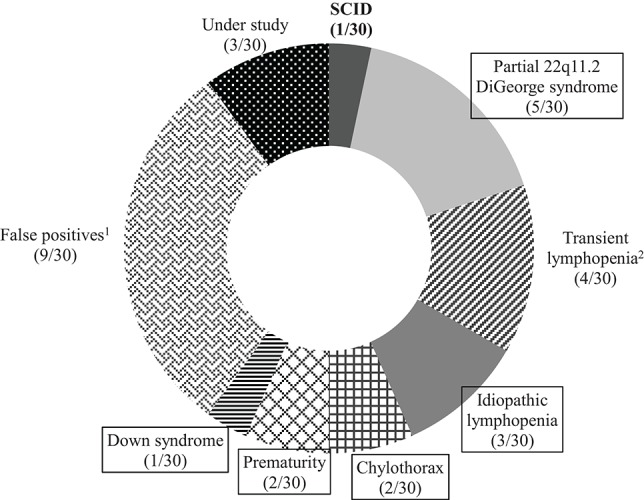
Final diagnoses in patients testing positive in Catalonia (2017–2018). ^1^False-positives due to initially normal lymphocyte count with normalization of TRECs between 3 and 6 months of life; ^2^Transient lymphopenia due to initially low lymphocyte count with recovery in the following months. Final diagnosis of the 13 non-SCID lymphopenic patients are now boxed. SCID, severe combined immunodeficiency.

Newborns with positive detection were referred to the SCID-CRU per protocol. The various diagnoses in the 30 patients were as follows (Figure 4): SCID (1/30), partial 22q11 DiGeorge syndrome (5/30), idiopathic lymphopenia (3/30), chylothorax (2/30), prematurity (2/30), and Down syndrome (1/30). Laboratory and clinical data of non-SCID TCL patients are described in Table 2. Nine patients were considered to have false-positive results (i.e., initially low TRECs and normal lymphocyte count, with normalization of TRECs between 3 and 6 months of life), four patients had transient lymphopenia (i.e., initially low TRECs and low lymphocyte count, with recovery in the following months), and three patients are currently under study (SCID excluded, but requiring additional TREC testing at 3 and 6 months of life). The incidence of clinically significant non-SCID TCL was 1 case in 10,069 newborns (43% of positive detections).

**Table 2:** Clinical and laboratory data in non-SCID TCL patients.

**Patient**	**Gestational age**	**Sex**	**Prenatal diagnosis**	**TREC levels (copies/μL)**	Lymphocyte count, (x10^**9**^/L)^*^	Lymphocyte subsets:T cells/B cells/NK cells (x10^**9**^/L)^**^	**Lympho-proliferationassay**	**Clinical features at birth**	**Diagnosis**
1	40	Female	No	17	4.2	2.1/19.9/1.1	Normal	None	22q11.2 DS
2	40	Female	No	5	5.4	1.6/1.1/2.3	Normal	None	22q11.2 DS
3	34	Female	Yes	5	2.6	1.1/0.6/0.7	Normal	Congenital heart disease	22q11.2 DS
4	39	Female	Yes	17	2	1.9/1.4/2	Normal	Congenital heart disease and hypocalcemia due to hypoparathyroidism	22q11.2 DS
5	41	Female	No	17	1.7	1.2/0.04/0.3	Normal	None	22q11.2 duplication
6	40	Male	No	14	3.7	2.2/0.6/0.6	Normal	None	Idiopathic lymphopenia
7	40	Male	No	7	3.3	1.7/0.6/0.7	Normal	None	Idiopathic lymphopenia
8	35.2	Male	No	6	1.7	2/0.2/0.2	Normal	None	Idiopathic lymphopenia
9	34	Female	Yes	6	7.8	NA	Normal	Congenital Chylothorax	Chylothorax
10	34	Female	Yes	4	7.4	NA	Normal	Hydrops fetalis	Chylothorax
11	32	Male	No	7	2.7	2.9/0.8/0.2	Normal	None	Prematurity
12	29	Male	No	18	4.4	2.9/1/0.2	Normal	None	Prematurity
13	31	Female	No	15	3	NA	Normal	Esophageal atresia	Down syndrome

* Reference value: 3.4–7.6;

** *Reference values: T-cells: 1.8–5.9; B-cells: 0.6–1.9; NK-cells: 0.1–1.3. CGH, comparative genomic hybridization; DS, deletion syndrome; NA, not available; NBS, newborn screening; SCID, severe combined immunodeficiency; TREC, T-cell receptor excision circle*.

Thus, the incidence of SCID was 1 in 130,903 newborns. The SCID patient was a Caucasian male of 35 weeks' gestation with 0/0 TREC copies/μL in DBS. Lymphocyte count was 0.4 × 10^9^/L with a T-B- NK+ phenotype and an absent *in vitro* proliferative response to mitogens. Whole exome sequencing was performed, but no causative mutations were found. However, a variant of uncertain significance was identified in the *LIG1*gene. The patient underwent HSCT at the age of 2 months of life using reduced intensity conditioning, with a good clinical outcome and immunological reconstitution.

## Discussion

This study reports the Catalonian NBS experience for SCID detection based on TREC quantification, and enables detection of non-SCID lymphopenic conditions in addition to SCID. After a 2-year experience and more than 130,000 newborns screened, one SCID patient and 13 other conditions were identified. In addition, we were able to consolidate our detection strategy and the characterization, diagnosis, and follow up protocol for positive cases.

As a pioneer initiative in Europe, we encourage the definition of a single technical approach for SCID newborn screening to facilitate future comparative studies between different countries. With this in mind, we decided to implement the commercially available EnLite Neonatal TREC kit (Perkin Elmer, Turku, Finland), which has been validated elsewhere and has shown comparable median TREC values between different studies (4, 6, 22, 23). This kit allows for DNA elution and gene amplification in a single process, as well as standardization and reproducibility according to ISO 15189 standards. With the aim of minimizing the previously described inter-lot variability (6), we established a retest cutoff higher than the request second sample cutoff, which provided a range between the retest and detection cutoffs. The initial retest cutoff was 34 copies/μL (3rd percentile), which was changed to 24 copies/μL (1st percentile) in 2018, whereas the request second sample cutoff was maintained at 20 copies/μL.

The initial retest cutoff of 34 copies/μL (3rd percentile) led to a retest rate of 3.34%, which was considered too high in comparison with data from other authors (24). Hence, we then evaluated the first percentile from our population (24 copies/μL) as a new retest cutoff. We found that 1,477 of 1,478 babies with initial TREC values between 34 and 25 copies/μL were normal: 1,455 (98.4%) after duplicate repetition and 22 (1.6%) after a second DBS analysis. Only one baby within this range was referred for study in the SCID-CRU and the diagnosis was not a case of SCID, the objective of this screening. Following this analysis, we decided that it was reasonable to switch to 24 copies/μL as the TREC repeat cutoff, implemented since 2018. That change reduced the repetition rate to 1.4%, now comparable to the rate described by others (24) and led to a reduction in the screening cost per patient.

Our internal algorithm showed a second sample request rate and positive detection rate of 0.2 and 0.02%, respectively, in keeping with the rates in previous studies, as summarized in the review by van der Spek et al. (24). In addition, the percentiles of our population corresponding to each cutoff are comparable to those of other diseases included in our program, such as congenital hypothyroidism, phenylketonuria, and other metabolic diseases. Of note, the method was unable to adequately quantify beta-actin copies in some of the external quality control samples from the CDC program, a shortcoming that may be related to PCR inhibition, as was suggested by the CDC itself. Nonetheless, this limitation did not affect the results of SCID screening.

Several studies have used combined TREC and KREC screening assays to simultaneously detect T- and B-cell defects (18, 25), including a pioneer study performed in Spain by de Felipe et al. (26), but we decided to focus on classical SCID patients who completely fulfill the NBS criteria, in accordance with the experience in the United States (2, 5). Although inclusion of KREC values to the TREC assay would enable the detection of patients with hypomorphic mutations leading to a leaky SCID phenotype and delayed-onset adenosine deaminase (ADA) deficiency (14, 25, 27), it might also result in an increase in false-positive testing and higher recall and retest rates.

Our data support the previously reported influence of gestational age and sex on TREC values (27–30). Thus, in the case of low values in preterm babies or low-birth-weight term newborns (i.e., TREC values above 5 copies/μL in preterm babies and 10 copies/μL in term newborns), cautious evaluation and a request for a second sample are needed at term and when birth weight reaches >2,500 g, respectively. To our knowledge, there is no explanation for the significantly higher TREC values seen in female newborns; however, these differences have been described previously in a cohort study reported by Rechavi et al. (4) Further studies may provide data to define the reason for these differences and their clinical implications, if any.

With regard to gestational age, median TREC levels in our cohort rose significantly from 28 to 32 weeks gestation in accordance with T-cell maturation in this period, a wider period of time than those reported by other authors (4, 18, 26) (Supplementary Material S4).

Overall, there were 30 positive detections in the 129,614 newborns screened (130,903 before applying the exclusion criteria). All were referred to the SCID-CRU per protocol. Nine patients were considered to have false-positive results due to initially normal total lymphocyte and CD3+ counts with normalization of TRECs between 3 and 6 months of life, whereas four others had transient lymphopenia at the beginning with recovery in the following months, and three are currently under study [none of the three patients under study met the SCID criteria, as defined by Kwan et al. (2)].

The diagnosis of the T-B-NK+ SCID patient permitted timely referral to a specialized treatment center for HSCT, after which T and B lymphocyte counts normalized with fully reconstituted function, eliminating the need for IgG supplementation at 1 year of age.

The incidence of SCID found in this study (1 in 130,903) is lower than the reported rates from other areas (2, 4, 24). However, we anticipate that a more robust incidence will be established when the population analyzed reaches 200,000 newborns. In this line, a retrospective study in our region demonstrated an incidence of classically diagnosed SCID of 1 in 57,000 newborns (unpublished data). In addition to the SCID patient, 13 infants with non-SCID TCL were identified, yielding an incidence of 1 in 10,069 newborns (43% positive detections). This is similar to the rate reported by others (31). The diagnostic distribution was also comparable. It is worth mentioning that early detection of patients with 22q11.2 deletion syndrome allowed a prompt intervention to anticipate complications, as reported by our group (Mol Genet Genomic Med, accepted).

Of note, one of the strengths of our SCID NBS approach is the psychological support offered to the families. A positive result in any NBS program is a cause of concern for parents at this vulnerable time because of an unfamiliarity with these illnesses, the young age of the patients, and the stress on families after a birth. Therefore, in neonates initially testing positive, a psychologist provided support starting from the first contact with the family in a joint visit with the pediatric immunologist. The psychologist's task was to guarantee that parents were able to understand and assimilate the information about the possible diagnosis and the process to follow in the event that it was confirmed. The family was offered a space for containment and support for the impact of the diagnosis, and strategies were activated to control anxiety. The psychologist maintained contact to ensure adherence to the treatments prescribed and compliance with the control visits and tests. Support was intensified in the most complicated moments, such as hospital admission and isolation, and parents were accompanied in all phases related to transplantation. Several cases of parental anxiety were detected and appropriately managed. In the yearly evaluation, families rated the psychological support in our NBS program very favorably.

The main limitation of this study is the relatively small number of newborns screened, although the similarity between our results and those reported in larger cohorts supports their validity. In addition, retrospective data show an incidence of SCID in our region similar to that of other Western European countries (6, 18) and the United States (5).

To conclude, TREC quantification in DBS for SCID detection has been satisfactorily implemented in the Catalonian NBS program. Retesting, requested second samples, and positive detection rates were optimal with the current algorithm and similar to published data. Our results provide further evidence to support the inclusion of SCID in NBS programs in other regions and countries. Longer follow-up is needed to define the exact incidence of SCID in Catalonia.

## Data Availability Statement

The datasets generated for this study are available on request to the corresponding author.

## Ethics Statement

The studies involving human participants were reviewed and approved by Comité Ético from Agència de Salut Pública (Catalonia). Written informed consent to participate in this study was provided by the participants' legal guardian/next of kin.

## Author Contributions

AA-R, AM-N, and PS-P designed the study. YQ and TC collected the data and participated in the analytical tools. AA-R and JM-S organized the database. AA-R and RL-G performed the statistical analysis. Results were analyzed and interpreted by AA-R, AR, AM-N, and PS-P. AA-R, AM-N, and PS-P wrote the first draft of the manuscript. MG-P, MM-G, JR, RC, SP-G, JG, JG-V, and JM-S wrote sections of the manuscript. All authors contributed to manuscript revision and read and approved the submitted version.

### Conflict of Interest

The authors declare that the research was conducted in the absence of any commercial or financial relationships that could be construed as a potential conflict of interest.
