# Supplementary material for: First Universal Newborn Screening Program for Severe Combined Immunodeficiency in Europe. Two-Years' Experience in Catalonia (Spain)
Source: Front Immunol. 2019 Oct 22;10:2406. doi: 10.3389/fimmu.2019.02406 (PMC6818460; doi:10.3389/fimmu.2019.02406)
Supplement: Supplementary file 1 [file Data_Sheet_1.pdf]

Supplementary Material S1. TREC assay validation

Summary of TREC assay variation during the initial method verification				
	SAMPLES			
	n	**Cal A**	**C1**	**C3**
**SD (copies/µL)**	28	0.74	0.38	0,44
**kit SD expected values**		<0.84	<0.65	<0.71
**CV (%)**	28	85	39	46
**kit CV expected values**		<101	<73	<81

	n	**Value**
**LoD (copies/µL)**	20	3.4
**LoQ (copies/µL)**	20	4.9
**External Quality assessment**	5	100%
**Sensitivity**	3006	100%
**Specificity**	3006	99.9%
**Contamination risk**	111	0.9%

Suppl. Mat. S1. Abbreviations: CV, coefficient of variation (%); DBS, dry blood spot; LoD, limit of Detection (copies/µL); LoQ, limit of Quantification (copies/µL); SCID, severe combined immunodeficiency; SD, standard deviation (copies/µL); TREC, T-cell receptor excision circle

- Calibrator A (Cal A), Control 1 and Control 2 were from lot 652072.
- Variation was expressed as standard deviations (SD) in the natural logarithmic (ln) scale and as CV% in lognormal scale; both were compared with the kit supplier's SD and CV% for the same range of values.
- LoD and LoQ calculated with a blank sample and very low TRECs sample analyzed during 10 days in duplicate and by calculating the critical value.
- External quality assessment was evaluated as a qualitative method (CDC Program, % of successful results are indicated).
- Sensitivity was evaluated using 6 DBS samples from babies with confirmed SCID
- Contamination risk was evaluated with the C2 control (0 copies/µL) and a blank paper (without sample). A result of >10 copies/µL was considered as contamination.

Supplementary Material S2. SCID criteria defined by Kwan et al (2)

	CD3 T Cells/μL	PHA proliferation	Supporting features
**Typical SCID**	< 300	< 10% of normal	Detectable maternal T cells in peripheral blood; proven deleterious defect(s) in a known SCID gene

frontiers

Supplementary Material S3. List of 323 primary immunodeficiency disease genes included in our panel

List of 323 primary immunodeficiency disease genes											
ACP5	C4BPB	CD55	CTSC	FERMT3	IL10RB	LAT	MVK	PIK3R1	RNF31	TRAF3IP2	TAP1
ACTB	C5	CD59	CXCR4	FOXN1	IL12B	LCK	MYD88	PLCG2	RORC	TREX1	TAP2
ADA	C6	CD70	CYBA	FOXP3	IL12RB1	LIG1	MYO5A	PMS2	RPSA	TRNT1	TAPBP
ADAM17	C4A	CD79A	CYBB	FPR1	IL17F	LIG4	NBN	PNP	RTEL1	TTC37	TAZ
ADAMTS13	C4B	CD79B	DCLRE1B	G6PC	IL17RA	LMNA	NBS1	POLE1	SAMHD1	TTC7A	TBK1
ADAR	C4BPA	CD81	DCLRE1C	G6PC3	IL17RC	LPIN2	NCF1	PRF1	SBDS	TYK2	TBX1
AICDA	C7	CD8A	DEPTOR	G6PD	IL18	LRBA	NCF2	PRKCD	SERPING1	UNC119	TCF3
AIRE	C8A	CEBPE	DGKE	G6PT1	IL1RN	LRRC8A	NCF4	PRKDC	SH2D1A	UNC13D	TCIRG1
AK2	C8B	CECR1	DKC1	GATA2	IL21	LYST	NEIL3	PSMB8	SH3BP2	UNC93B1	TCN2
AKT1	C8G	CFB	DNMT3B	GFI1	IL21R	MAGT1	NFAT5	PSTPIP1	SKIV2L	TERC	TNFSF6
AP3B1	C9	CFD	DOCK2	GIMAP5	IL2RA	MALT1	NFKB1	PTPRC	SLC11A1	TERT	TRAC
AP3D1	CARD11	CFH	DOCK8	GP1BA	IL2RG	MAP3K14	NFKB2	RAB27A	SLC29A3	TFRC	TRAF3
AP4E1	CARD14	CFI	ELANE	HAX1	IL36RN	MASP1	NFKBIA	RAC2	SLC35C1	THBD	UNG
APOL1	CARD9	CFP	ELF4	ICOS	IL7R	MASP2	NHEJ1	RAG1	SLC37A4	TICAM1	USB1
ARPC1B	CARMIL2	CHD7	EPG5	IFIH1	INO80	MBL2	NHP2	RAG2	SLC46A1	TINF2	VPREB1
ATM	CASP10	CIITA	F12	IFNGR1	IRAK4	MCM10	NLRC4	RASGRP1	SMARCAL1	TLR3	VPS13B
BLM	CASP8	CLEC7A	FADD	IFNGR2	IRF7	MCM4	NLRP12	RASGRP2	SP110	TMC6	VPS45
BLNK	CD19	CLPB	FAM105B	IGHA1	IRF8	MEFV	NLRP3	RBCK1	SPINK5	TMC8	WAS
BLOC1S6	CD247	COH1	FAS	IGHG2	ISG15	MKL1	NOD2	RECQL4	STAT1	TMEM173	WASF2
BTK	CD27	COLEC11	FASLG	IGHM	ITCH	MLPH	NOP10	RFX5	STAT2	TNFAIP3	WIPF1
C1QA	CD3D	COPA	FCGR1A	IGLL1	ITGB2	MMACHC	NRAS	RFXANK	STAT3	TNFRSF11A	WRAP53
C1QB	CD3E	CORO1A	FCGR2A	IKBA	ITK	MPO	ORAI1	RFXAP	STAT5B	TNFRSF13B	XIAP
C1QC	CD3G	CR2	FCGR2B	IKBKB	JAGN1	MRE11A	PARN	RMRP	STIM1	TNFRSF13C	XRCC4
C1R	CD3Z	CSF2RA	FCGR3A	IKBKG	JAK2	MS4A1	PAX5	RNASEH2A	STK4	TNFRSF1A	ZAP70
C1S	CD40	CSF3R	FCGR3B	IKZF1	JAK3	MSH6	PGM3	RNASEH2B	STN1	TNFRSF4	ZBTB24
C2	CD40LG	CTLA4	FCGRT	IL10	KRAS	MSN	PIGA	RNASEH2C	STX11	TNFRSF6	ZNF345
C3	CD46	CTPS1	FCN3	IL10RA	LAMTOR2	MTHFD1	PIK3CD	RNF168	STXBP2	TNFSF12	

Supplementary Material S4. Median TREC copy numbers at each gestational week

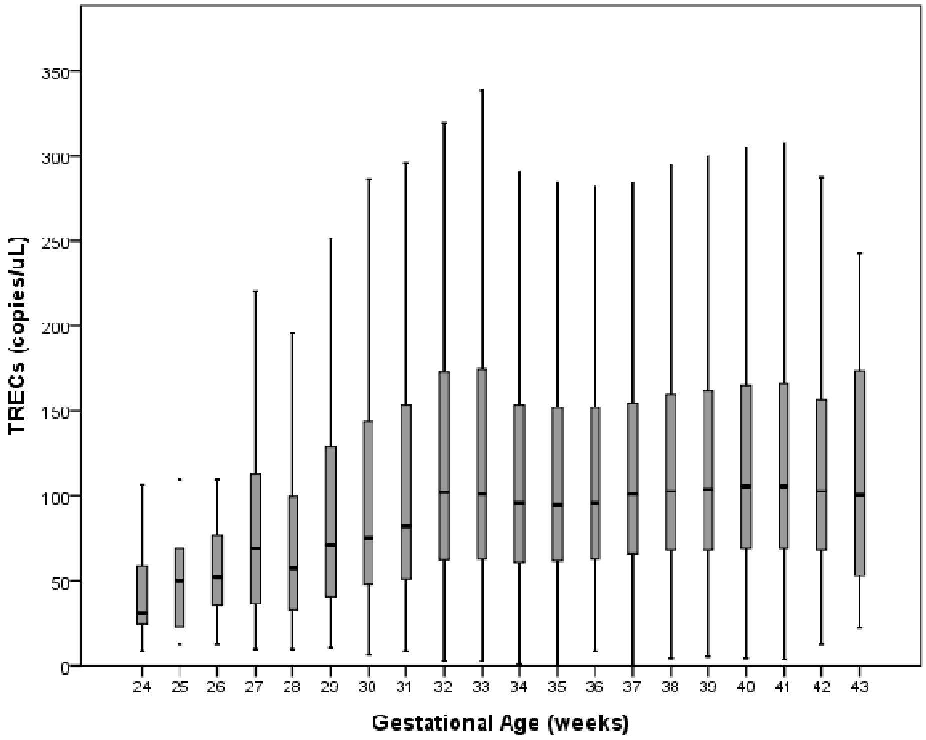

Suppl. Mat. S5. Error bars indicate 25th and 75th percentile TREC copy numbers at each gestational week. Median TREC levels in our cohort rose significantly between 28 and 32 weeks gestation, in accordance with T-cell maturation in this period, a wider period of time than those reported by other authors (4,18,26).

Abbreviations: TRECs: T-cell receptor excision circles

Supplementary Material S5. Flow cytometry protocols

Peripheral whole blood (50 µL) was incubated with a mix of specific conjugated monoclonal antibodies (mAb) from each panel and gently mixed for 20 min at room temperature (RT) in the dark. The composition of mAb, florochromes, and brands are specified in the following table:

**T B NK populations**		
Cluster of Differentiation	*Fluorochrome*	*Brand*
CD45	FITC	Beckman Coulter
CD4	RD1	Beckman Coulter
CD8	ECD	Beckman Coulter
CD3	PC5	Beckman Coulter
CD56	RD1	Beckman Coulter
CD19	ECD	Beckman Coulter
**CD45 R0/RA**		
Cluster of Differentiation	*Fluorochrome*	*Brand*
CD45RA	FITC	Becton Dickinson
CD45R0	PE	Becton Dickinson
CD3	ECD	Beckman Coulter
CD4	PerCP	Becton Dickinson
CD8	PE-Cy7	Beckman Coulter
**HLA-DR**		
Cluster of Differentiation	*Fluorochrome*	*Brand*
CD3	ECD	Beckman Coulter
CD4	APC	Cytognos
CD8	PE-Cy7	Beckman Coulter
HLA-DR	Pacific Blue	Beckman Coulter

Samples were treated with 1 mL of VersaLyse lysing solution (Beckman Coulter), vortexed, and incubated for 15 min at RT in the dark. Samples were then washed with phosphate-buffered saline (PBS), stored at RT in the dark, and analyzed within 1 h. Samples were acquired with a Navios EX Flow Cytometer (Beckman Coulter), equipped with three lasers: a 405-nm violet laser, a 488-nm blue laser, and a 638-nm red laser. At least 100,000 events were acquired from each sample. Flow cytometry data were analyzed with Kaluza software (Beckman Coulter).
